# Variability of protein level and phosphorylation status caused by biopsy protocol design in human skeletal muscle analyses

**DOI:** 10.1186/1756-0500-4-488

**Published:** 2011-11-10

**Authors:** Marc-André Caron, Steve J Charette, François Maltais, Richard Debigaré

**Affiliations:** 1 Centre de recherche, Institut Universitaire de Cardiologie et de Pneumologie de Québec, Université Laval, Québec, Canada; 2 Institut de biologie intégrative et des systèmes, Pavillon Charles-Eugène-Marchand, Université Laval, Québec Canada

## Abstract

**Background:**

Bergström needle biopsy is widely used to sample skeletal muscle in order to study cell signaling directly in human tissue. Consequences of the biopsy protocol design on muscle protein quantity and quality remain unclear. The aim of the present study was to assess the impact of different events surrounding biopsy protocol on the stability of the Western blot signal of eukaryotic translation initiation factor 4E binding protein 1 (4E-BP1), Akt, glycogen synthase kinase-3β (GSK-3β), muscle RING finger protein 1 (MuRF1) and p70 S6 kinase (p70 S6K). Six healthy subjects underwent four biopsies of the *vastus lateralis*, distributed into two distinct visits spaced by 48 hrs. At visit 1, a basal biopsy in the right leg was performed in the morning (R1) followed by a second in the left leg in the afternoon (AF). At visit 2, a second basal biopsy (R2) was collected from the right leg. Low intensity mobilization (3 × 20 right leg extensions) was performed and a final biopsy (Mob) was collected using the same incision site as R2.

**Results:**

Akt and p70 S6K phosphorylation levels were increased by 83% when AF biopsy was compared to R1. Mob condition induced important phosphorylation of p70 S6K when compared to R2. Comparison of R1 and R2 biopsies revealed a relative stability of the signal for both total and phosphorylated proteins.

**Conclusions:**

This study highlights the importance to standardize muscle biopsy protocols in order to minimize the method-induced variation when analyzing Western blot signals.

## Background

Skeletal muscle atrophy is a common clinical component of numerous diseases including AIDS, cancer, chronic heart failure, chronic obstructive pulmonary disease, and diabetes [1-3]. Muscle atrophy has a wide spectrum of consequences for chronically ill patients, ranging from an aggravated morbidity to a seriously impaired survival prognostic [4,5]. Since there is currently no effective treatment for atrophy [6], there is a crucial need for a clear comprehension of muscle depletion at the molecular level.

Fundamentally, muscle mass maintenance relies on a tight regulation of protein synthesis and degradation, two fundamental processes influenced by numerous signaling pathways. The phosphatidylinositol-3 kinase/Akt pathway has been pointed out as a key coordinator of synthesis and degradation. Akt, the central protein of this pathway, is an upstream kinase to several targets implicated in both processes [7,8]. With the discovery of the muscle-specific E3 ligases Muscle RING finger 1 (MuRF1) and Muscle Atrophy F-box in 2001 [9], the ubiquitin-proteasome pathway has also emerged as an important putative player in the atrophying process. Historically, these two pathways have been predominantly investigated in cells and animal models. However, to ascertain consistency with human muscle biology, these pathways have been repeatedly studied directly in human samples [10,11].

Human muscle samples required for biochemical studies have been obtained repeatedly with a Bergström biopsy needle [12]. A concern with this technique is the potential introduction of variability in studied parameters because of sampling site selection. Furthermore, protein levels and/or their associated activities (kinase or ligase) in muscle tissue are affected by stimuli such as exercise [13] and nutritional state [14]. In the context of studies aimed at investigating cell signaling in muscle tissue, it cannot be excluded that sampling site variation combined to unstandardized nutritional and physical activity status could impact data analysis.

Western blotting is broadly used to dissect cell signaling from muscle samples obtained by needle biopsies. This technique provides precious pieces of information on protein content or phosphorylation levels in a given sample. As cell signaling is a vast and complex domain, optimal confidence in results obtained by Western blotting is essential to appropriately answer experimental questions and to accurately angle future research hypothesis. Thus, the aim of this study was to document the impact of experimental conditions surrounding human *vastus lateralis *biopsy procedures on the variability of the Western blot signals. Both total and phosphorylated forms of key proteins of the PI3K/Akt (i.e. Akt, eukaryotic translation initiation factor 4E binding protein 1 [4E-BP1], Glycogen synthase kinase-3 beta [GSK-3β], p70 S6 kinase [p70 S6K]) and the ubiquitin-proteasome (i.e. MuRF1) pathways were investigated. Specifically, the present study was designed to answer three questions: 1) What is the intrinsic variability induced by the Bergström needle biopsy on muscle cell signaling when successive biopsies are performed on separate days? 2) Is performing skeletal muscle biopsies at different periods in a given day induces variability in the signal detection? 3) Does a low intensity mobilization, such as walking or stair climbing, induces variability in the signal detection? To properly answer these questions, a specific sequence of four muscle biopsies was designed. First, two identical basal biopsies, spaced by 48 hours, were performed in the morning in rested and fasted subjects (R1 and R2). Next, a biopsy was performed in the afternoon (AF) of the first day after R1. Finally, the fourth biopsy was taken right after a low-intensity mobilization (Mob), which was performed immediately after the basal state biopsy (R2). These time points likely represent common daily circumstances faced by investigators when human muscle biopsies is being included in the research protocol. We hypothesized that the combined effects of a variable period of the day or light mobilization will induce variability in the Western blot signals in total and phosphorylated proteins when compared to a rested and fasted state.

## Methods

### Subjects and Ethics Statement

Six healthy males aged between 26 and 45 years volunteered to participate in the study. These individuals were free of any medication. All subjects were moderately active (3-5 hrs of physical activity/week) and the body mass index of the cohort was 23.5 ± 1.1 (mean ± SEM). The Clinical Research Ethics Committee of the "Institut Universitaire de Cardiologie et de Pneumologie de Québec" approved the study protocol and each patient signed a written informed consent form.

### Study design

The study protocol required two distinct visits, each including two biopsies. At the first visit, subjects arrived at the laboratory between 8 and 9 AM after a 12-hours fasting period. Subjects had to rest for 15 min and were questioned about their activities in the last 24 hours. This interview was performed to ensure that no sustained exercise period occurred before the protocol. The first biopsy of the right *vastus lateralis *was performed under local anesthesia (2cc lidocaine 2%) using the Bergström method [12], 15 cm above the patella, as routinely done in our laboratory [15]. This biopsy was identified as "rested and fasted-1" (R1). Subjects were then invited to resume their typical daily activities without any food restriction until 2 PM, when they had to return for the next sample collection. Knowing that muscle biopsies can be performed at any time of the day, depending on the availability of the research subject and the medical staff, we avoid any restriction in feeding and mobilization after the first biopsy (R1). This format allowed us to collect the second biopsy (AF) and to answer the second research question of this study. Subjects rested for 15 min and the second needle biopsy was performed on the left *vastus lateralis*, 15 cm above the patella. The second visit took place 48 hours later. As for the initial visit, subjects arrived between 8 and 9 AM after a 12-hours fasting period and rested for 15 min. The third needle biopsy was then performed on the right *vastus lateralis*, 3 cm above the first biopsy site and away from the healing tissue. This biopsy was identified as "rested and fasted-2" (R2). Immediately after this biopsy, a temporary bandage was applied on the site of incision and subjects had to perform 3 sets of 20 leg extensions while a 5-kg weight was applied over the distal end of the right leg. The fourth biopsy was collected immediately after this local mobilization through the same incision that was used for the third one. This biopsy was identified as "acute mobilization and fasted" (Mob) and allowed us to answer the third question of this study. Elapsed time between third and fourth biopsies was less than 15 minutes. After each biopsy, muscle specimens were cleaned with an absorbent tissue to remove blood before being snap frozen in liquid nitrogen and kept at -80°C for future analyses.

### Muscle protein extraction

To ensure maximal blood contaminant removal from samples, approximately 50 mg of muscle was placed in 1 ml ice-cold PBS 1X and lightly agitated by hand for 45 seconds. The muscle specimen was recovered and immediately placed in 500 μl ice-cold lysis buffer [5 mM Tris-HCl pH 8.0, 1 mM EDTA, 1 mM EGTA, 1% glycerol, 1 mM β-mercaptoethanol, protease inhibitor cocktail set III (EMD biosciences), phosphatase inhibitor cocktails 1 and 2 (Sigma-Aldrich)] and homogenized on ice with a Polytron^® ^homogenizer. The resulting extract was centrifuged (13 000 rpm, 4°C, 5 min) and the supernatant was transferred to a new tube. An aliquot was reserved for Bradford protein assay and Laemmli buffer was added to the extract. Protein extract was then boiled for 10 min, aliquoted and kept at -80°C for further analyses.

### Western blotting

To control for the non-linear relationship between protein quantity and Western blot signal, we loaded on each gel serial quantities of a standardized protein extract, in order to create a calibration curve, as suggested by Charette et al [16]. The standardized protein extract was obtained from 90% confluent human primary myoblats of a healthy subject. These cells were washed once in ice-cold PBS before being scrapped in 300 μl Laemmli buffer. Cell extract was then boiled for 10 min and kept at -80°C. Western blots were performed in duplicate with 10-30 μg total proteins using standard SDS-PAGE procedures. Following transfer onto nitrocellulose membrane, blotting was completed with the following antibodies from Cell Signaling Technology (Danvers, MA, USA): anti-phospho-4E-BP1 (#2855, 1:1000), anti-Akt (#9272, 1:2000), anti-phospho-Akt (#9271S, 1:1000), anti-GSK-3β (#9315, 1:1000), anti-phospho-GSK-3β (#9331, 1:1000), anti-MuRF1 (#4305S, 1:1000), anti-p70 S6K (#9202, 1:1000), anti-phospho-p70 S6K (#9206S, 1:1000). Proteins of interest were detected using a secondary antibody coupled to horseradish peroxidase (#7074, #7076, 1:5000, Cell Signaling Technology). To ensure equal loading, every result was normalized to tubulin (T5168, 1:20 000, Sigma-Aldrich, St-Louis, MO, USA).

### Western blot analysis

**Figure 1:**
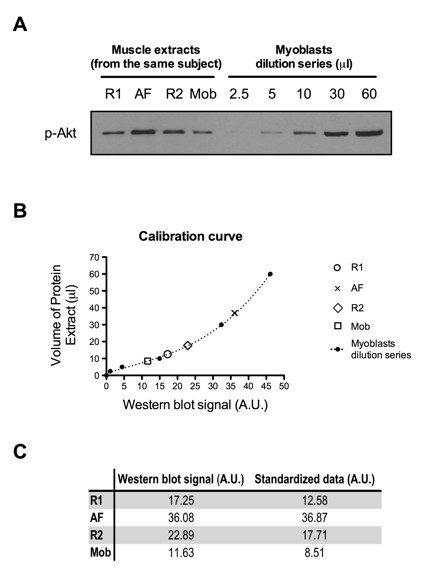
**Western blot analysis strategy exemplified for phospho-Akt**. A) To control for the non-linearity between Western blot signal and the amount of protein loaded, a dilution series of protein extracts obtained from human primary myoblasts was loaded on every gel in order to build a calibration curve. B) Each volume of myoblasts protein extract was plotted against their optical density. Optical densities of subject's muscle extracts were then reported on the calibration curve and the corresponding volume of protein extract was found. C) These data were considered the standardized values and were used for the final analysis. p-Akt = phospho-Akt

Specific protein abundance and phosphorylation levels were analyzed as illustrated in Figure 1. For a given subject, his four muscle extracts were loaded onto a gel, along with a dilution series of the protein extract obtained from human myoblasts (Figure 1, panel A). Densitometry of the resulting bands was obtained using ImageJ software [17]. Using data from the dilution series, a calibration curve was plotted (protein extract volume against Western blot signal) and the Western blot signal obtained for all the biopsies of a given individual were reported on this curve as illustrated in Figure 1, panel B. The corresponding volume of protein extract for each Western blot signal was determined and considered as standardized data, as shown in Figure 1, panel C. To control for protein loading, standardized data were reported on tubulin Western blot signal and these corrected values were used for subsequent comparative analyses.

### Statistical analysis

Data are presented as mean ± standard error of the mean (SEM). R1 was set as the referential value and absolute variations of either AF or R2 samples were reported against this condition. These comparisons were performed to assess the repeatability of the measure (question 1: R1 vs R2) and the impact of feeding and daily activities (question 2: R1 vs AF) on cell signaling. A sub-analysis was also performed, where R2 was set as the referential value and compared to Mob condition, in order to measure the impact of a local acute mobilization on protein expression and activity levels as evidenced by phosphorylation level variations (question 3). In all analyses, each subject was used as its own control. The intrinsic variability produced by the entire Western blot technique was evaluated by calculating the mean standardized signal variation of every duplicate performed for this study. By using this method, a technique-induced variability of 32% on the measure was found. The inter-measures concordance was evaluated by performing Pearson correlations between Western blot signals for Akt, GSK-3β, MuRF1 and p70 S6K using R2 and Mob biopsies. *P*-value < 0.05 was considered statistically significant.

## Results

### Total protein amount variation

**Figure 2:**
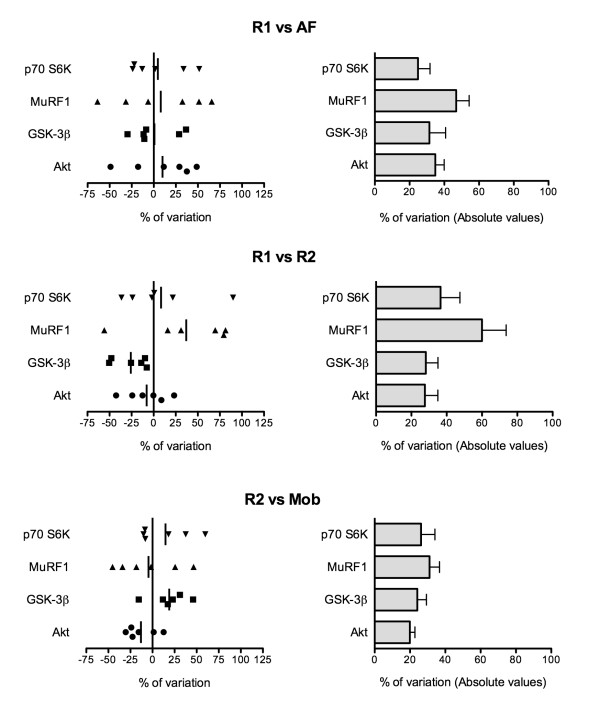
**Muscle biopsy conditions induce minimal to moderate variability on total proteins Western blot signal**. All biopsies of each subject were blotted against Akt, GSK-3β, MuRF1 and p70 S6K. Standardized value for every band optical density was obtained and protein expression level between biopsy conditions were compared. All results were normalized with tubulin signal to ensure equal loading. Data are expressed in percentage of variation between two biopsy situations. On the left side, data are expressed as real values where positive and negative variation levels are depicted. The vertical line represents the mean variation level. On the right side, every difference between compared biopsies was transformed in absolute values, so that no distinction was made between an increase and a decrease in protein expression between two conditions. Results are expressed as mean ± SEM of 6 intra-subject variations.

The impact of the conditions surrounding biopsy procedures on total protein amount was evaluated by measuring the intra-subject variation of the Western blot signal obtained with each sampling conditions (R1, AF, R2, Mob). In our hands, the technique-induced variability was assessed to be 28% for the 4 total proteins tested (Table 1). Two separate analyses were performed with each protein. First, as depicted on the left side of Figure 2, relative differences in Western signal between biopsy conditions are expressed as actual mathematical variations. Thus, positive and negative variations are reported for all six subjects. In order to abrogate the effect of combining positive and negative values in mean calculation and to reflect the proper fluctuation induced by sampling conditions, absolute variation values were also calculated. Results are presented on the right side of Figure 2. Using this second analysis, GSK-3β protein level was found to be the most stable, with a range of variation between 24% for rest vs acute mobilization conditions (R2 vs Mob) and 31% for rest and fasted vs active and fed conditions (R1 vs AF). On the opposite, MuRF1 was the most fluctuating protein, reaching a maximal variation level of 60% over two biopsies taken in similar conditions 48 hours apart (R1 vs R2). For all four conditions compared, Akt signal variations were approximately 30%. In a similar way, p70 S6K protein level variations ranged from 25% (R1 vs AF) to 37% (R1 vs R2). In all studied proteins, signal comparison of both biopsies taken at rest and in fasted state (R1 vs R2) revealed a range of variation between 28% (Akt and GSK-3β) and 60% (MuRF1). Similarly, a global analysis of all the protein levels uncovered a variation ranging between 25% (p70 S6K) and 47% (MuRF1) for biopsies taken at two selected time points during a given day (R1 vs AF). When comparing signals obtained with two successive biopsies (R2 and Mob), the variation recorded was constantly one of the lowest in all four tested proteins, ranging from 20% (Akt) to 31% (MuRF1).

**Table 1:** Western blot technique-induced variability of the duplicates per protein.

Protein tested	Technique-induced variability
Akt	27%
phospho-Akt	25%
GSK-3β	26%
phospho-GSK-3β	25%
p70 S6K	20%
phospho-p70 S6K	50%
MuRF1	37%
phospho-4E-BP1	48%
***Total proteins***	**28%**
***Phospho-proteins***	**37%**
***Overall***	**32%**

4E-BP1, eukaryotic translation initiation factor 4E binding protein 1; GSK-3β, Glycogen synthase kinase-3 beta; p70 S6K, p70 S6 kinase; MuRF1, Muscle RING finger 1.

Western blot signal concordance obtained with two successive muscle biopsies (R2 and Mob) was assessed using Pearson correlations. Strong positive correlations were found for Akt (r = 0.8045), GSK-3β (r = 0.9435) and p70 S6K (r = 0.8551) whereas the correlation was moderate in the case of MuRF1 (r = 0.6059).

### Phosphorylation state variation

**Figure 3:**
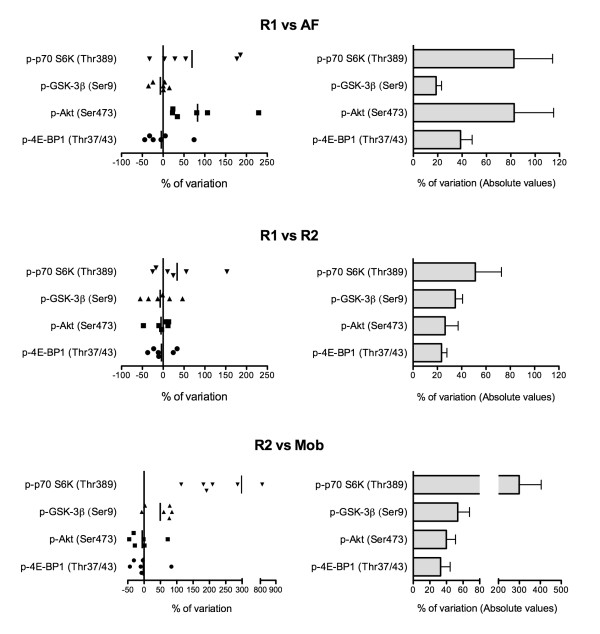
**Akt, GSK-3β, 4E-BP1 and p70 S6K phosphorylation levels are influenced by muscle biopsy conditions**. All biopsies of each subject were blotted against phospho-Akt, phospho-GSK-3β, phospho-4E-BP1, phospho-p70 S6K. Standardized value for every band optical density was obtained and protein expression level between biopsy conditions were compared as shown in bar graphs. All results were normalized with tubulin signal to ensure equal loading. Data are expressed in percentage of variation between two biopsy situations. On the left side, data are expressed as real values where positive and negative variation levels are depicted. The vertical line represents the mean variation level. On the right side, every difference between compared biopsies was transformed in absolute values, so that no distinction was made between an increase and a decrease in protein expression between two conditions. Results are expressed as mean ± SEM of 6 intra-subject variations. p- = phosphorylated

In a second set of analyses, we tested the impact of muscle sampling conditions (rest, activity/feeding and acute mobilization) on the phosphorylation state of key proteins related to muscle mass homeostasis. In our hands, the technique-induced variability was assessed to be 37% for the 4 phosphorylated proteins tested (Table 1). As presented with total proteins, phosphorylated proteins were analyzed both with actual and absolute values and the results are shown in Figure 3 (left and right side of the figure, respectively). As depicted on the right side of the figure, Western blot signal variability of phosphorylated Akt ranged from 26% between both rest and fasted conditions (R1 vs R2) to 83% between rest and fasted vs activity and fed conditions (R1 vs AF). GSK-3β and 4E-BP1 phosphorylation levels reached respectively variations of 19% (R1 vs AF) to 54% (R2 vs Mob) and 23% (R1 vs R2) to 39% (R1 vs AF). Phosphorylation state of p70 S6K reached a variation level of 299% when the acute mobilization (Mob) signals were compared to the rest and fasted condition (R2). Global analysis of the results reveals that R1/R2 comparison induced fluctuations of the signal ranging from 23% (phospho-4E-BP1) to 51% (phospho-p70 S6K). A spectrum of variation, ranging from 19% (phospho-GSK-3β) to 83% (phospho-Akt and phospho-p70 S6K), was found when the signals of activity and fed (AF) and rest and fasted (R1) conditions were compared. The protein phosphorylation comparisons of the second rest and fasted (R2) to the acute mobilization (Mob) conditions revealed variations ranging from 32% (phospho-4E-BP1) to 299% (phospho-p70 S6K). Finally, when analyzing the data expressed in actual values (Figure 3, left side), activity and fed condition (AF) exclusively induced positive Akt phosphorylation changes when Western blot signals were compared to R1. Similarly, acute mobilization condition (Mob) exclusively induced positive changes in p70 S6K phosphorylation state when signals were compared to the second rest and fasted condition (R2).

## Discussion

This study provides a quantitative measurement on the impact of experimental conditions when multiple Bergström needle biopsies are performed to study cell signaling in human muscle tissue using Western blotting. As other laboratory techniques, Western blot exhibits an inherent variability that is difficult to precisely evaluate. However, using triplicata of a given sample on a single gel, it has been estimated that Western blotting alone produces a coefficient of variation of approximately 10% [18]. Since assessment in triplicata implies that the same protein extract is used, the reported 10% variation does not take into account the additional variability that could be induced by protein extraction protocol and dosage. In our hands, the technique-induced variability was higher with phosphorylated proteins and the overall variability was assessed to be 32% (Table 1). Our duplicates were run onto two distinct gels instead of being on the same gel, likely explaining the higher variation in this study compared to the one previously cited [18]. This intrinsic 32% variation level was used as a guideline to weigh against the subsequent variation levels obtained in the present study.

Different patterns of variation were observed when total protein was considered. First observation, GSK-3β was found to be almost insensitive to muscle biopsy conditions. A second pattern was observed with Akt and p70 S6K. With a maximal variation level reaching 35%, total Akt exhibited a low amplitude response to every sampling conditions tested in the present protocol. Akt is a central kinase and interacts with an impressive number of partners [19]. This implies that at every moment, numerous intra- and extra-cellular signals regulate its kinase activity level. Thus, in a context of extensive regulation, a ~30% variation in the measurement of the total form of Akt is minimal, especially when assuming that the technique induced variability is 32% in our hands. This result possibly reflects the need of a strong stimulus, like sepsis for instance, to induce a noticeable regulation of Akt quantity [20,21]. Similarly total p70 S6K signal was not extensively affected by biopsy conditions. A third variation pattern was observed with MuRF1 protein. Moderate fluctuations reaching 31 to 60% in response to acute mobilization and activities were measured. These values are up to two fold higher than the variation level associated with the technique itself. Except for its induction in atrophied muscle [9,22], the regulation of MuRF1 is still unclear. Nonetheless, a difference of 60% in the signal of MuRF1 between two biopsies taken in similar conditions (R1 and R2) demonstrates that minor modifications in subject behavior can have a significant impact on its expression level. Strict and fast protein quantity regulation can be observed with particular proteins such as hypoxia inducible factor-1 alpha, the latter being rapidly marked and degraded by the ubiquitin-proteasome pathway in presence of oxygen (half-life < 5 min) [23]. MuRF1 accumulation is increased by energy deprivation conditions [24] and hyperinsulinemia [25]. Since subjects were fasted prior to the first biopsy (R1) and allowed to feed and move before the biopsy performed in the afternoon of the same day (AF), it might be argued that nutritional status could explain the variation of 47% observed in this particular situation. However, since positive and negative regulations are observed between R1 and AF biopsies, other unknown factors are probably influencing its level. MuRF1 being a key component of the ubiquitin-proteasome pathway with specific targeted proteins [26], it is conceivable that its level constantly fluctuates over time depending on the surrounding pool of intracellular proteins to be degraded.

In muscle tissue, protein phosphorylation is a rapid and well-described cellular response to numerous stimuli including inflammation [27], exercise [28] and nutrition [14]. Given the relative unsteadiness of protein phosphorylation levels, a wide range of variations was expected with phosphorylated protein measurement in this study. Even though Akt phosphorylation was relatively stable in most conditions, a variation of 83% between fasted and fed conditions (R1 vs AF) was observed. This situation is in agreement with the increased phosphorylation of Akt observed in skeletal muscle when systemic insulin is administered [29]. Considering the experimental design, it is difficult to discriminate between the feeding state and daily activities since neither was controlled between both R1 and AF biopsies. Nevertheless, this observation highlights the notion of standardizing the biopsy protocol in order to minimize fluctuation in the signal due to sampling technique when multiple biopsies are needed in the research protocol. Based on these considerations and as detailed in this study, we emphasize on the notion that the conditions surrounding muscle sampling in a given study should be clearly reported in the method section of every publication.

Mobilization was also found to be an important phosphorylation modulator of phospho-p70 S6K and, to a lesser extent, of phospho-GSK-3β. Since the prescribed mobilization in this study was of relatively low intensity for young healthy subjects, the impact of mobilization (stairs or walking) for elderly and/or diseased patients has the potential to create a similar impact as the one observed in the current protocol. Based on our results, it is clear that physical activity, even of a low level, should be avoided before sampling muscle tissue if a study is designed to analyze basal phosphorylation signals.

Interestingly, the two highest inter-biopsy variations found in the present study (phospho-Akt, R1 vs AF; phospho-p70 S6K, R2 vs Mob) were the only comparisons where all subjects uniformly responded with an increase in phosphorylation level to the stimulus. Since the interventions were of relatively low intensity, this implies that any physiological divergent event in a given protocol has the potential to alter muscle protein signaling and to induce false positive or negative results in Western blot based analysis. On the other hand, comparison of the two basal conditions (R1 and R2) systematically emerged as one of the most stable for all phosphorylated proteins tested in this study. This indicates that, despite the relative instability of protein phosphorylation state, taking simple precautions can circumvent unsolicited situations as observed for phospho-Akt and phospho-p70 S6K.

As this study was performed with human subjects, a small number of methodological considerations were taken into account when planning the protocol. Because Bergström needle biopsy is a relatively invasive technique, we choose to perform the afternoon biopsy (AF) on the left *vastus lateralis *in order to minimize subject's discomfort, given the fact they already underwent a biopsy procedure on the right leg the very same morning (R1 biopsy). The impact of leg change on protein variation is unknown. However, results obtained when comparing R1 and AF biopsies did not particularly fluctuate in terms of amplitude when put side by side to the other comparisons done for the same protein. This observation suggests that the change of sampled leg did not, by itself, induce a major bias, although the real impact cannot be clearly measured. Also, the second basal biopsy (R2) was collected approximately 3 cm over the first basal (R1) and it could be speculated that an inflammatory response occurred at the second sampling site in reaction to the first injury. Nevertheless, no macroscopic observation leads us to recognize that such a phenomenon was occurring at the time of the R2 biopsy. Despite our efforts to reduce subject discomfort, we experienced some difficulties in recruiting volunteers to undertake such an invasive trial. The net result of the low number of subjects was a decreased sensitivity to detect variability between sampling conditions. Finally, it cannot be excluded that a healing or trauma effect was occurring between R2 and Mob biopsies. Although the exact effects of these processes on post-transcriptional modifications are unknown, this could explain in part the differences found in the phosphorylation levels of p70 S6K and GSK-3β between these two conditions.

## Conclusion

In conclusion, this study clearly demonstrates that unstandardized human muscle sampling protocols can alter the final answer obtained when Western blotting is performed to measure a specific response. This statement is particularly true when protein phosphorylation is the signal being assessed. Our data suggest that phosphorylation of Akt and p70 S6K might be affected by nutritional status and activity. Accordingly, scientists investigating these proteins in muscle biopsies should be aware of these findings during design of the study and interpretation of the results, particularly in situations in which changes in protein level/phosphorylation are of lower magnitude. Finally, it cannot be excluded that other phosphorylated proteins or other post-translational processes (glycosylation, methylation) could react in a similar manner when unstandardized sampling protocol is used.

## Competing interests

The authors declare that they have no competing interests.

## Authors' contributions

MAC participated in the design of the study, performed all the experimental work and data analysis. He also drafted the manuscript. SJC participated in the design of the study and helped to draft the manuscript. FM and RD were involved in the experimental design, coordinated the study and supervised the manuscript redaction. All authors read and approved the manuscript.
